# Protofibrils of Amyloid-β are Important Targets of a Disease-Modifying Approach for Alzheimer’s Disease

**DOI:** 10.3390/ijms21030952

**Published:** 2020-01-31

**Authors:** Kenjiro Ono, Mayumi Tsuji

**Affiliations:** 1 Department of Internal Medicine, Division of Neurology, School of Medicine, Showa University, Tokyo 142-8666, Japan; 2 Department of Pharmacology, School of Medicine, Showa University, Tokyo 142-8666, Japan; tsujim@med.showa-u.ac.jp

**Keywords:** Alzheimer’s disease, amyloid β-protein (Aβ), mAb158, oligomers, protofibrils

## Abstract

Worldwide, Alzheimer’s disease (AD) is the most common age-related neurodegenerative disease and is characterized by unique pathological hallmarks in the brain, including plaques composed of amyloid β-protein (Aβ) and neurofibrillary tangles of tau protein. Genetic studies, biochemical data, and animal models have suggested that Aβ is responsible for the pathogenesis of AD (i.e., the amyloid hypothesis). Indeed, Aβ molecules tend to aggregate, forming oligomers, protofibrils, and mature fibrils. However, while these Aβ species form amyloid plaques of the type implicated in AD neurodegeneration, recent clinical trials designed to reduce the production of Aβ and/or the plaque burden have not demonstrated clinical efficacy. In addition, recent studies using synthetic Aβ peptides, cell culture models, Arctic transgenic mice, and human samples of AD brain tissues have suggested that the pre-fibrillar forms of Aβ, particularly Aβ protofibrils, may be the most critical species, compared with extracellular fibrillar forms. We recently reported that protofibrils of Aβ_1-42_ disturbed membrane integrity by inducing reactive oxygen species generation and lipid peroxidation, resulting in decreased membrane fluidity, intracellular calcium dysregulation, depolarization, and synaptic toxicity. Therefore, the therapeutic reduction of protofibrils may prevent the progression of AD by ameliorating neuronal damage and cognitive dysfunction through multiple mechanisms.

## 1. Introduction

Neurodegenerative diseases, such as Alzheimer’s disease (AD), Parkinson’s disease, and spinocerebellar ataxia, have characteristic abnormal protein aggregates in the brain. In AD, the two neuropathological characteristics are amyloid plaques composed of amyloid β-protein (Aβ) and neurofibrillary tangles of hyperphosphorylated tau protein [1].

Human genetic association studies, biochemical analyses of AD plaque content, and various animal models with altered Aβ or tau expression have strongly implicated Aβ and tau in AD pathogenesis [1]. Furthermore, many in vivo and in vitro studies have demonstrated the neurotoxicity of these amyloidogenic proteins. However, amyloid neurotoxicity depends strongly on Aβ’s primary structure and aggregation state. For example, two predominant Aβ forms are produced in humans and are comprised of either 40 (Aβ_1-40_) or 42 (Aβ_1-42_) amino acid residues. The relative proportion of Aβ_1-42_ appears to be particularly crucial for AD progression, as this longer form is more prone to aggregation and is inherently more toxic than Aβ_1-40_ [2]. Aβ molecules form low molecular weight (LMW) oligomers, high molecular weight (HMW) oligomers such as protofibrils (PFs), and mature fibrils, which have been suggested to be primary agents of neuronal dysfunction in AD [3]. Although these Aβ aggregates may directly cause neuronal injury by acting on synapses or indirectly by activating astrocytes and microglia [2], evidence also supports the hypothesis that soluble oligomeric Aβ plays an important role in AD pathogenesis (i.e., the oligomer hypothesis) [1,3,4].

Many types of oligomeric Aβ species have been demonstrated in vitro, with PFs being commonly described. Aβ PFs are defined as curved linear structures >100 kDa that remain soluble upon centrifugation at 16,000–18,000× g [3,5,6,7]. The neurotoxicity of these Aβ PFs formed in vitro, as well as their ability to induce electrophysiological effects on neurons, has been demonstrated by several groups [8,9,10,11]. Arctic Aβ is the result of a mutation in the gene that encodes the amyloid precursor protein (APP) and leads to the production of a particular Aβ species, [Glu22Gly]Aβ, with a high propensity to form PFs [12]. We recently reported that PFs disturb membrane integrity by inducing reactive oxygen species’ (ROS) generation and lipid peroxidation, resulting in decreased membrane fluidity, intracellular calcium dysregulation, depolarization, and impaired long-term potentiation (LTP). In addition, the damaging effects of PFs were found to be significantly greater than those of LMW-Aβ_1-42_ [13].

Current treatments for AD are primarily aimed at mitigating symptoms, while disease-modifying approaches are aimed at halting or attenuating the progression of the disease, such as inhibiting Aβ production and aggregation or promoting Aβ_1-42_ clearance [14]. However, despite many long and expensive trials, no disease-modifying drug for AD has been approved [15,16]. A recent failure in phase 3 involved the investigation of a β secretase in patients with mild-to-moderate AD [17]. Other large, phase 3 trials using anti-amyloid approaches including semagacestat [18], bapineuzumab [19], and solanezumab [20], have yielded disappointing results. However, it has been recently reported that BAN2401 (mAb158), an antibody developed for early AD with a unique target binding profile selective for Aβ PFs, significantly slowed cognitive decline by 30%, with a concomitant reduction in amyloid plaques, compared with placebo at 18 months [21].

In this review, we focus on recent developments from basic and clinical studies of PFs, including research findings from our laboratory.

## 2. PFs Are Primary Toxins in AD

### 2.1. The Discovery of PFs and Their Role in AD Pathogenesis

PFs were first described by Teplow and colleagues in 1997 [6]. Using a size exclusion chromatography (SEC) system and the synthetic Aβ_1-42_ peptide, they found a peak representing a large (>100 kDa) soluble species before the peak of the LMW-Aβ (mainly monomer) [6]. Using electron microscopy (EM), they further revealed that this peak contained predominantly curved fibrils, with a diameter of ~5 nm and a length of up to 200 nm, which they termed PFs [6]. Subsequently, the authors elucidated that the PFs were composed primarily of β-sheets and partially random coils and α-helices in a secondary structure [6]. In the same year, using atomic force microscopy (AFM), Lansbury’s group found the existence of a metastable intermediate species, which was termed Aβ PF [22]. Many data have shown that LMW-Aβ oligomers are on-pathway to fibril formation, while HMW-Aβ oligomers such as PFs are off-pathway [22,23,24,25]. Although the PF-to-fibril transition, characterized by PF elongation, was very slow, preformed fibrillar seeds greatly accelerated this conversion [22]. Recently, using a combination of high-speed AFM with thioflavin T assay, EM, and re-injection assays by SEC, we demonstrated that fibril formation from PFs is more difficult than that from LMW-Aβ, suggesting that mature fibrils of Aβ_1-42_ are primarily formed from LMW-Aβ_1-42_ and not from PFs [24]. Furthermore, we determined that PFs instead supplied precursors to LMW-Aβ_1-42_ by their dissociation, suggesting that PFs may not always represent the “on-pathway” of Aβ_1-42_ aggregation from the monomer to the mature fibrils [24]. Kodali and Wetzel mentioned that, although Aβ_1-40_ PFs can grow by monomer addition, their rate of growth is lower than that of mature fibrils. Additionally, while Aβ_1-40_ monomer was able to support the extension of mature fibrils at low concentrations of, Aβ_1-40_ PFs exhibited no extension [23]. They suggested another terminology, “curvilinear fibrils”, for the description of off-pathway PFs instead of PFs as on-pathway precursors of fibrils [23]. It was recently revealed that curvilinear fibrils inhibit fibril formation not only by slowing fibril nucleation and elongation, but also by actively disrupting either process based on combined thioflavin kinetics and AFM imaging data [26]. On the other hand, Iwatsubo’s group showed that Aβ_1-42_ PF injection induced Aβ deposition in the brains of A7 mice overexpressing human APP695 and harboring the K670N, M671L, and T714I familial AD neuronal mutations, suggesting that Aβ PFs may act as a seed for Aβ aggregation in vivo [27]. The injection of Aβ PFs mixed with apoE3 significantly attenuated Aβ deposition, whereas apoE4 did not, suggesting that the suppressive effect of apoE3 on the structural conversion of Aβ PFs to fibrils is stronger than that of apoE4, thereby impeding Aβ deposition in vivo [27].

### 2.2. PFs Are Primary Toxins in AD

**Figure 1:**
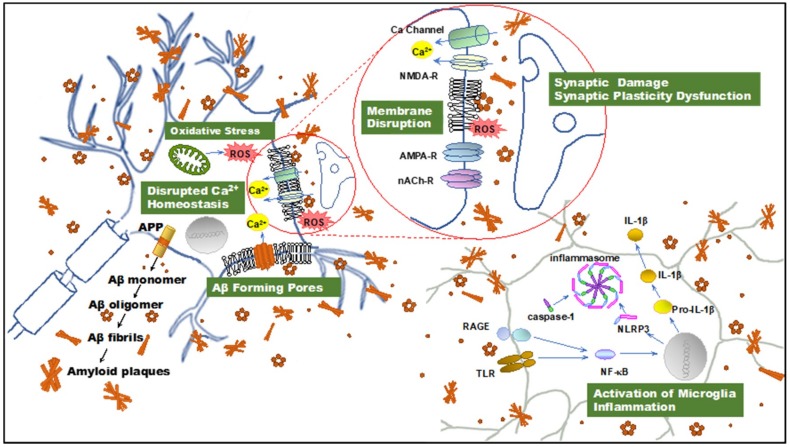
Illustration summarizing amyloid β-protein (Aβ) neurotoxicity. Aβ aggregates induce disruption of cellular homeostasis, which may be the result of inducing or exacerbating membrane disruption, oxidative stress, calcium dysregulation, synaptic plasticity dysfunction, and inflammation. APP: amyloid precursor protein; Aβ: amyloid β-protein; ROS: reactive oxygen species; NMDAR, *N*-methyl-d-aspartate receptor. AMPAR, α-amino-3-hydroxy-5-methyl-4-isoxazolepropionic acid receptor; nAChR, nicotinic acetylcholine receptor; TLR: toll-like receptor; RAGE: receptor for advanced glycation endproducts; NF-κB, nuclear factor κB; NLRP3: NOD-, LRR- and pyrin domain-containing protein 3; IL-1β: interleukin-1β.

The solubility and diffusible nature of soluble oligomers may render them more effective in terms of intra- and extra-cellular interactions and engaging microglial receptors compared with mature insoluble fibrils. Indeed, it has been demonstrated that astrocytes engulf large amounts of accumulated, rather than digested, Aβ_1-42_ PFs. This intracellular storage of Aβ_1-42_ results in severe astrocytic endosomal/lysosomal defects and the secretion of extracellular vesicles containing N-truncated, neurotoxic Aβ [28]. Aβ_1-42_ PFs have also been shown to induce an inflammatory process through microglial activation [29] and initiate Toll-like receptor (TLR) signaling (Figure 1) [30]. In addition, these PFs are preferentially internalized by microglia [31]. Furthermore, it has been reported that Aβ_1-42_ PFs are more effective at inducing microglial tumor necrosis factor α (TNFα) production in BV-2 and primary murine microglia in vitro than monomers and mature fibrils. Moreover, PFs of Aβ_1-40_ exhibit significantly less activity than concentration-matched Aβ_1-42_ [29]. Aβ_1-42_ PFs also have been shown to trigger a time- and myeloid differentiation protein (MyD) 88-dependent process that generates TNFα and interleukin-1β (IL-1β) mRNA, along with pro and mature forms of the intracellular IL-1β protein [30]. The accumulation of both IL-1β forms has indicated that Aβ_1-42_ PFs are able to prime and activate the Nod-like receptor (NLR) P3 inflammasome. In this process, Aβ has been shown to elicit a quantized burst of secreted IL-1β which occurs prior to the Aβ priming of the microglia. The IL-1β secretion burst appears to be rapid and not sustained, yet it may be re-initiated with additional Aβ stimulation. These findings indicate multiple modes of IL-1β regulation by Aβ_1-42_ PFs, including TLR/MyD88-mediated priming, NLRP3 inflammasome activation, and modulation of the IL-1β secretory process, suggesting wide-ranging effects of Aβ on the innate immune response [30]. 

Recent evidence has suggested that the neuronal cell membrane is the chief site of oligomer-mediated neuronal damage. We recently studied the cellular response to short exposures to PFs using multiple indices of membrane integrity, cytolysis, oxidative stress, and synaptic function. We found that cellular membrane and metabolic integrity were more severely disrupted by PFs of Aβ_1-42_ than LMW-Aβ_1-42_, as evidenced by various experimental systems, including cell viability and leakage assays, fluorometric measures of ROS generation, lipid peroxidation assays, and electrophysiological recordings [13]. While our results for lactate dehydrogenase (LDH) and calcein and ethidium homodimer-1 assays reflected cellular membrane damage by PFs of Aβ_1-42_ to a greater extent than LMW-Aβ_1-42_, 3-[4,5-dimethylthiazol-2-yl]-2,5-diphenyltetrazolium bromide metabolism (MTT) and water soluble tetrazolium (WST) assays reflecting mitochondrial enzyme activity, they demonstrated only small differences between the Aβs in different cellular models, including SH-SY5Y cells and a healthy, human-induced pluripotent stem line [13]. From these results, in terms of short-term Aβ_1-42_ PF treatment, Aβ_1-42_ PFs may first attack the cell membrane, followed by subsequent damage to the mitochondria, although Aβ_1-42_ dimers might not be removed clearly in LMW-Aβ_1-42_ preparation using the above-mentioned SEC method [6]. Next, we found that exposure to PFs of Aβ_1-42_ in SH-SY5Y cells induces more severe oxidative stress, including greater levels of ROS production and membrane lipid peroxidation, than LMW-Aβ_1-42_. Indeed, many studies have reported that oxidative stress, which occurs in the presence of a physiological imbalance between ROS generation and antioxidant capacity, is a critical pathogenic mechanism in AD progression [32]. Along with the direct destruction/modification of lipids, DNA, and proteins, the byproducts of lipid peroxidation produced during oxidative stress cause damage to the mitochondria and upregulate tau phosphorylation, which appears essential for NFT formation [33]. In addition, the generation of superoxide by Aβ aggregates may lead to mitochondrial impairment and further induce ROS generation, thereby establishing a positive feedback pathway that ultimately results in cell death [34]. Moreover, Aβ aggregates may directly interact with the mitochondrial respiratory chain, causing metabolic dysfunction and increased ROS production [35]. In our study, the PFs of Aβ_1-42_ also reduced neuronal membrane fluidity to a significantly greater extent than LMW-Aβ_1-42_. Thus, we consider the possibility that the effects on membrane fluidity, and the resulting neuronal damage, depend on the specific Aβ conformation [13].

We further demonstrated that short exposures to Aβ_1-42_ PFs induces higher concentrations of [Ca^2+^]_i_ than LMW-Aβ_1-42_, whereas a reduced depolarization-induced [Ca^2+^]_i_ influx through voltage-dependent Ca^2+^ channels was observed following longer exposures to Aβ_1-42_ PFs [13]. These results suggested that PFs may not only directly damage voltage-gated calcium channels for a short time, but also alter the cell membrane environment required for proper channel insertion or gating for longer periods, as evidenced by lipid peroxidation and membrane fluidity measurements [13]. 

Consistent with the changes observed in [Ca^2+^]_i_ and the loss of membrane integrity, the application of PFs of Aβ_1-42_, but not those of LMW-Aβ_1-42_, also has been shown to depolarize SH-SY5Y cells and significantly reduce membrane input resistance [13]. Bode et al. monitored transmembrane currents during Aβ exposure at the extracellular face of excised membranes from HEK293 cells, and found that annular Aβ_1-42_ oligomers formed ion channels, whereas Aβ_1-40_ oligomers and mature fibrils and monomers did not [36]. Drolle et al. used multi-component lipid models to mimic healthy and AD states of neuronal membranes and posited that Aβ_1-42_ increases lipid membrane roughness and membrane conductance, possibly through pore formation [37]. Taken together, the [Ca^2+^]_i_ increase evoked by PFs may be due to pore formation and oxidative damage, as well as the suppression of calcium egress and sequestration pathways secondary to metabolic disruption. 

We also demonstrated that Aβ_1-42_ PFs significantly inhibit LTP formation in the mouse hippocampal CA1 subfield [13]. Similarly, it has been reported that PFs induce electrophysiological changes, including rapid increases in the excitatory post synaptic and action potentials, membrane depolarizations in rat cortical neurons [8], and the inhibition of LTP in the rat hippocampus [38]. Excessive ROS accumulation and decreased membrane fluidity are associated with effects on LTP and learning [39,40]. Furthermore, membrane pore formation may also impair cellular and synaptic functions (Figure 1) [41,42]. 

The small (35kDa) and highly dispersible protein, secreted-frizzled-related protein 1 (SFRP1), regulates transmembrane metalloprotease ADAM10 activity and is essential for the development of tissue homeostasis and constitutive levels of α-secretase in the brain [43]. As a novel player in AD pathogenesis, SFRP1 has been shown to be significantly increased in the brain and cerebrospinal fluid of patients with AD. In addition, SFRP1 has been demonstrated in human AD cases and mouse models to prevent Aβ PF formation by binding to Aβ, suggesting it may be a promising AD therapeutic target [44].

### 2.3. Arctic Mutation Causes Aβ PF Formation

Arctic mutation is a pathogenic APP mutation located within the Aβ sequence at codon 693, at which point glutamic acid is substituted for glycine (E693G). In 2001, Lannfelt’s group named the mutation the ‘Arctic’ mutation because the family in which it was detected was from northern Sweden [12]. Affected subjects have clinical features of early AD and plasma levels of both Aβ_1-40_ and Aβ_1-42_ are lower in mutation carriers compared with healthy family members. In addition, concentrations of Aβ_1-42_ were found to be reduced in media from cells transfected with APP_E693G_ [12]. Furthermore, the authors reported that the Arctic Aβ mutation (Aβ_1-40_ Arc) causes enhanced the formation of Aβ_1-40_ PFs in vitro [12]. Subsequently, Lannfelt’s group found that the Arctic mutation significantly accelerated Aβ_1-42_ PF formation, as well as PF fibrillization [7]. 

It has been reported that Aβ_1-40_ Arc inhibits LTP ~100-fold more potently than wild-type Aβ_1-40_ when wild-type and Aβ_1-40_ Arc peptides are injected into the CA1 area in rats intracerebroventricularly. In this study, the isolated soluble fraction that included the PFs of Aβ_1-40_ Arc after high-speed centrifugation was shown to still retain full LTP inhibitory activity [38]. In a later study, Lord et al. demonstrated that the Arc mutation accelerates early intraneuronal Aβ aggregation and PF formation, followed by plaque formation, in APP transgenic mice with both the Arctic (E693G) and Swedish (K670N, M671L) mutations (tg-APP_ArcSwe_) [45,46]. In addition, cognitive deficits have been shown to occur concomitantly with the formation of intracellular Aβ deposits, but before plaque formation, in transgenic mice [45]. In addition, the levels of PFs in the brain, but not those of total Aβ, have been correlated with spatial learning, which adds further evidence to the theory that soluble PFs are the toxic species [47]. The pool of toxic Aβ species reportedly consists of molecules in the size range of 80 to 500 kDa [48].

## 3. Therapeutic Approaches Targeting Aβ PFs

### 3.1. Small Molecules Inhibit the Formation of Aβ PFs

Small molecules with the potential to mitigate toxic AD species such as Aβ_1-42_ PFs are promising preventive and therapeutic candidates. We previously demonstrated that a grape-seed-derived polyphenol was able to inhibit Aβ_1-42_ aggregation by preventing PF formation, pre-protofibrillar oligomerization, and random coil-aggregation-prone α-helix/β-sheet secondary structure transitions using various analyses, including circular dichroism spectroscopy, thioflavin T fluorescence, SEC, and EM [49]. Importantly, this polyphenol demonstrated protective effects in cytotoxicity assays, in which it was mixed with Aβ_1-42_ aggregates and exposed to cells [49]. Furthermore, our in vivo studies using the Tg2576 AD mouse model showed that this grape seed polyphenolic extract significantly attenuated AD-type cognitive deterioration and reduced cerebral amyloid deposition [50]. 

Using multiple molecular dynamics (MD) simulations, Jin et al. reported that dihydrochalcone, a compound extracted from the daemonorops draco tree, could effectively inhibit Aβ_1-42_ fibrillization and reduce Aβ-induced cytotoxicity by destabilizing the Aβ PFs. In this process, dihydrochalcone was shown to bind to the cavity of the Aβ_1-40_/Aβ_1-42_ PFs themselves and disrupt the D23-K28 salt bridge and inter-peptide β–sheet in the β1 region [51]. In addition, Zhou et al. reported that 1,2-(dimethoxymethano)fullerene (DMF), a water-soluble fullerene derivative, strongly inhibited Aβ_1-42_ aggregation by binding with Aβ PFs on three dominant binding sites, namely, the central hydrophobic core (17LVFFA21), the turn site (27NKGAI31), and the C-terminal β-sheet site comprised of glycine and hydrophobic residues (31IIGLMVGGVVI41), by MD stimulations [52]. In addition, the binding of DMF to the turn region served to disrupt the D23-K28 salt-bridge critical for PF Aβ fibril formation [52]. Another series of MD stimulations showed that wgx-50, a compound extracted from the Sichuan pepper (Zanthoxylum bungeanum), can destabilize Aβ_1-42_ PFs through three possible stable binding sites, including two sites in the hydrophobic grooves on the surface of the Aβ PFs, which resulted in no significant changes in Aβ structure, and one site in the interior that caused PF destabilization. At this site, wgx-50 was observed to be packed against the side chains of I32 and L34, disrupting the D23-K28 salt bridge and partially opening the two tightly compacted β-sheets [53]. Recently, Saini et al. reported that a resveratrol and clioquinol hybrid compound, (E)-5-(4-hydroxystyryl)quinolone-8-ol, inhibits Aβ_1-42_ aggregation by preventing the conformational transition of the Aβ_1-42_ monomer and causing destablization of the Aβ_1-42_ PF structure using MD simulation [54]. The destabilizing mechanisms of the Aβ_1-42_ PF structure may be due to the increasing interchain distance between chains A–B, disrupting the salt-bridge interaction between D23-K28 and decreasing the number of backbone hydrogen bonds between the chains [54]. In the same year, it was reported that β-sheet breaker peptides, particularly PPFFE pentapeptides, display strong destablizing effects that shift the energy minima toward the lowest value of sheet content and the lowest number of hydrogen bonds in Aβ_1-42_ PFs, using in silico methodologies including the molecular mechanics Poisson–Bolzmann surface area method and MD simulations [55].

### 3.2. Aβ PF-Selective Antibody

PFs have been identified in the human brain and the APP transgenic mouse brain [48,56]. mAb158 is a murine monoclonal antibody developed to selectively target HMW-Aβ_1-42_ assemblies [56]. Using an enzyme-linked immunosorbent assay (ELISA), it has been elucidated that mAb158 has an at least 1000-fold higher selectivity for PFs than monomeric Aβ and 10-15 times better binding affinity to PFs than to mature fibrils, thereby targeting the more toxic species of the peptide [57]. In immunohistochemistry, mAb158 also detects Aβ in plaques and the vasculature of AD brains because of the massive amount of Aβ in these structures [58]. In addition, Lord et al. reported that mAb158 inhibits in vitro Aβ_1-42_ fibril formation and protected cells from Aβ PF-induced cytotoxicity [59]. A co-culture study of astrocytes, neurons, and oligodendrocytes exposed to Aβ_1-42_ PFs in the presence or absence of mAb158 demonstrated that the presence of mAb158 almost entirely abolished Aβ accumulation in astrocytes, indicating an effect towards Aβ PF degradation. Consequently, mAb158 treatment was shown to rescue neurons from Aβ-induced cell death [60].

The treatment of tg-APP_ArcSwe_ mice with mAb158 resulted in the prevention of plaque formation if the antibody was administered before the appearance of plaques in young mice. If the treatment was started later in this mouse model, levels of insoluble Aβ were unaffected in the brains of plaque-bearing older mice. However, in both cases, soluble Aβ PF levels were diminished, supporting the notion that mAb158 can selectively reduce PF levels [59]. Similarly, the authors found that PF levels were elevated in young tg-APP_ArcSwe_ mice compared with several transgenic models lacking the Arctic mutation. In older tg-APP_ArcSwe_ mice with plaque deposition, the levels of Aβ PFs were approximately 50% higher than in younger mice, whereas levels of total Aβ were exponentially increased. Young tg-APP_ArcSwe_ mice showed deficits in spatial learning, and individual performances in the Morris water maze were inversely correlated with levels of Aβ PF, but not with total Aβ levels. These findings indicated that Aβ PFs accumulated in an age-dependent manner, and increased levels of Aβ PFs may result in spatial learning impairments in tg-APP_ArcSwe_ mice [47]. Lannfelt et al. reported that the murine version of mAb158 reached the brain and reduced brain PF levels by 42% in an exposure-dependent manner both after long-term (13 weeks) and short-term (4 weeks) treatment in tg-APP_ArcSwe_ mice [14]. Notably, a 53% reduction in PFs/oligomers in the cerebrospinal fluid (CSF), found to be correlated with reduced brain PF levels, was observed after long-term treatment, suggesting that CSF PFs/oligomers may be used as potential biomarkers of AD [14].

Recently, Sehlin’s group succeeded in facilitating the brain uptake of mAb158 by using transferrin receptor-mediated transcytosis across the blood–brain barrier in tg-APP_ArcSwe_ mice [61]. ELISA analysis of the brain extracts demonstrated a 40% reduction in soluble Aβ PFs in both ten-fold lower-dose modified mAb158 and high-dose mAb158-treated mice, whereas there was no Aβ PF reduction in mice treated with a low dose of mAb158 [61]. Furthermore, ex vivo autoradiography and PET imaging have revealed different brain distribution patterns of modified mAb158 (brain parenchyma) and mAb158 (central periventricular areas), suggesting that these antibodies may affect Aβ levels by different mechanisms. This strategy may allow for decreased antibody doses, thereby reducing the side effects and treatment costs [61].

### 3.3. Clinical Application of mAb158

BAN2401, a humanized IgG1 monoclonal form of mAb158, exhibits a strong binding preference for soluble Aβ PFs compared with monomers [14]. In addition, it has been confirmed that both mAb158 and BAN2401 efficiently immunoprecipitate soluble Aβ aggregates in human AD brain extracts.

The first clinical study of BAN2401 demonstrated that the compound was safe and well tolerated in mild to moderate AD [62]. The incidence of amyloid-related imaging abnormalities (ARIA-E for edema /H for hemorrhage) on brain MRI scans was comparable to that of the placebo. BAN2401 exposure was approximately dose-proportional, with a serum terminal elimination half-life of approximately seven days. Only a slight increase in plasma Aβ_1-40_ was observed, but there were no measurable effects of BAN2401 on CSF biomarkers such as Aβ_1-42_, total-tau, and phosphorylayed-tau (p-tau) [62]. A recent phase 2 randomized trial reported that BAN2401’s highest dose (10 mg/kg) significantly slowed cognitive decline in early AD, with a concomitant reduction in amyloid plaques, as measured by amyloid PET compared with placebo at 18 months [21]. BAN2401 significantly reduced amyloid plaques in the brain at all five treatment doses used in the trial, which involved 856 patients with mild cognitive impairment. The 30% slowing of cognitive decline at 18 months was based on the Alzheimer’s Disease Composite Score (ADCOMS) created by Eisai. On the more widely used Alzheimer’s Disease Assessment Scale cognitive subscale (ADAS-Cog), the highest dose of BAN2401 slowed a cognitive decline of 47% compared with placebo. However, the trial was not large enough to definitively demonstrate efficacy in improving cognitive function according to an overall optimistic statement from the Alzheimer Association. The drug also did not achieve its primary efficacy endpoint, namely, a change from baseline on the ADCOMS at 12 months [21]. Currently, BAN2401 is a part of an ongoing phase 3 clinical trial. In contrast, other clinical trials of monoclonal antibodies targeting fibrillar Aβ, such as bapineuzumab [63], or soluble monomeric Aβ, such as solanezumab [20], have failed to produce clinical effects.

In the fall of 2019, after trials of the drug EMERGE (aducanumab; BIIB037) were previously discontinued following a phase III futility analysis, Biogen, the company that developed the drug, announced that subsequent analysis of a larger dataset instead showed that EMERGE had met its primary endpoint. Patients on the highest dose, 10 mg/kg, had a significant reduction in decline in terms of the primary endpoint using the Clinical Dementia Rating Scale-Sum of Boxes (CDR-SB). This group also declined less in terms of secondary endpoints, including the Mini-Mental State Examination (MMSE), ADAS-Cog, and the Alzheimer’s Disease Cooperative Study/Activities of Daily Living scale adapted for patients with mild cognitive impairment (ADCS-ADL-MCI). In a parallel clinical trial of aducanumab, termed the ENGAGE trial, aducanumab did not meet the primary endpoint; however, an exploratory analysis suggested that a subgroup of people who had received 10 or more 10 mg/kg doses declined more slowly, which is consistent with the EMERGE participants. In both trials, aducanumab caused a dose-dependent reduction in brain Aβ and CSF p-tau. Based on the updated data analysis, Biogen announced plans to apply for regulatory approval of aducanumab in the US in early 2020 [64]. Since aducanumab may also bind aggregates such as oligomers of Aβ [65], these results may be important for interpreting data from the phase 3 clinical trial of BAN2401.

## 4. PFs Are Present in Other Neurodegenerative Diseases

PFs are formed from proteins implicated in other neurodegenerative diseases, including tauopathy [66], Parkinson’s disease [67,68], familial amyloid polyneuropathy [69], and Huntington’s disease [70], indicating a common mechanism. Similar to Aβ, tau and α-synuclein (αS) also form PFs with annular, pore-like structures, thereby exerting membrane permeabilization activity [66,67]. Analyses of annular tau PFs in brain tissue from patients with progressive supranuclear palsy, as well as that from the P301L mouse model, indicated that the annular PFs of tau are preceded by tau oligomers and do not go on to form neurofibrillarly tangles (mature fibrils) [66]. In addition, it was recently reported that the αS oligomer and PFs interconvert during polymerization reactions, using the thioflavin T assay combined with SEC and EM [68]. Similarly, Groenning et al. described a dynamic transthyretin (TTR) protofibril structure that exchanges protomers with highly unfolded monomers in solution, using a combination of primarily small-angle X-ray scattering and hydrogen exchange mass spectrometry analysis. The TTR PFs were shown to only grow to an approximate final size of 2900 kDa and a length of 70 nm [69]. In a recent micro electron diffraction study at 0.75Å resolution, ultrahigh-resolution cryo-EM revealed that prion PFs are stabilized by a dense three-dimensional network of stabilizing hydrogen bonds that link residues between and within its β strands through polar clasps [71].

## 5. Conclusions and Future Perspectives

Unlike current therapies limited to the treatment of AD symptoms, research on Aβ aggregation has rapidly advanced, with growing evidence that soluble pre-fibrillar aggregates (i.e., oligomers of Aβ) are proximate neurotoxins. Indeed, recent data from both in vitro and in vivo studies have suggested that HMW oligomers as PFs induce neuronal injury and cognitive deficits via multiple mechanisms, including not only increasing Aβ plaque accumulation but also increasing direct membrane and synaptic damage. Furthermore, additional projects to fully characterize the PFs actually present in the human brain have been undertaken. Aβ PFs may be the primary pathogenic species of Aβ-related cognitive deficits, particularly in the early stage of AD, although it remains to be established how Aβ PFs, alone or together with other soluble oligomeric Aβ species, cause the neurodegeneration leading to AD. Disease-modifying therapies targeting toxic PFs will reach the clinical stage in the near future, and may have the potential to delay or even halt the further progression of AD. Further clarification of the toxic PFs of brain Aβ should aid in the development of more effective and safe drugs, as well as in novel diagnostic assays.

## Abbreviations

Aβ	amyloid β-protein
AD	Alzheimer’s disease
ADAS-Cog	Alzheimer’s Disease Assessment Scale cognitive subscale
ADCOMS	Alzheimer’s Disease Composite Score
AFM	atomic force microscopy
APP	amyloid precursor protein
ARIA	amyloid-related imaging abnormalities
αS	α-synuclein
CSF	cerebrospinal fluid
DMF	1,2-(dimethoxymethano)fullerene
ELISA	enzyme-linked immunosorbent assay
EM	electron microscopy
HMW	high molecular weight
IL-1β	interleukin-1β
LDH	lactate dehydrogenase
LMW	low molecular weight
LTPs	long-term potentiation
MD	molecular dynamics
MTT	3-[4,5-dimethylthiazol-2-yl]-2,5-diphenyltetrazolium bromide metabolism
MyD	myeloid differentiation protein
NLR	Nod-like receptor
PFs	protofibrils
p-tau	phosphorylayed-tau
ROS	reactive oxygen species
SEC	size exclusion chromatography
SFRP1	secreted-frizzled-related protein 1
TLR	Toll-like receptor
TNFα	tumor necrosis factor α
TTR	transthyretin
WST	water soluble tetrazolium
